# The Genetic Basis of Host Preference and Resting Behavior in the Major African Malaria Vector, *Anopheles arabiensis*

**DOI:** 10.1371/journal.pgen.1006303

**Published:** 2016-09-15

**Authors:** Bradley J Main, Yoosook Lee, Heather M. Ferguson, Katharina S. Kreppel, Anicet Kihonda, Nicodem J. Govella, Travis C. Collier, Anthony J. Cornel, Eleazar Eskin, Eun Yong Kang, Catelyn C. Nieman, Allison M. Weakley, Gregory C. Lanzaro

**Affiliations:** 1 Vector Genetics Laboratory, Department of Pathology, Microbiology and Immunology/University of California, Davis, Davis, California, United States of America; 2 Institute of Biodiversity, Animal Health and Comparative Medicine, University of Glasgow, Glasgow, United Kingdom; 3 Environmental Health and Ecological Sciences Group, Ifakara Health Institute, Ifakara, United Republic of Tanzania; 4 Department of Entomology and Nematology, University of California, Davis, Davis, California, United States of America; 5 Department of Computer Science, University of California, Los Angeles, Los Angeles, California, United States of America; Vanderbilt University, UNITED STATES

## Abstract

Malaria transmission is dependent on the propensity of *Anopheles* mosquitoes to bite humans (anthropophily) instead of other dead end hosts. Recent increases in the usage of Long Lasting Insecticide Treated Nets (LLINs) in Africa have been associated with reductions in highly anthropophilic and endophilic vectors such as *Anopheles gambiae s*.*s*., leaving species with a broader host range, such as *Anopheles arabiensis*, as the most prominent remaining source of transmission in many settings. *An*. *arabiensis* appears to be more of a generalist in terms of its host choice and resting behavior, which may be due to phenotypic plasticity and/or segregating allelic variation. To investigate the genetic basis of host choice and resting behavior in *An*. *arabiensis* we sequenced the genomes of 23 human-fed and 25 cattle-fed mosquitoes collected both in-doors and out-doors in the Kilombero Valley, Tanzania. We identified a total of 4,820,851 SNPs, which were used to conduct the first genome-wide estimates of “SNP heritability” for host choice and resting behavior in this species. A genetic component was detected for host choice (human vs cow fed; permuted *P* = 0.002), but there was no evidence of a genetic component for resting behavior (indoors versus outside; permuted *P* = 0.465). A principal component analysis (PCA) segregated individuals based on genomic variation into three groups which were characterized by differences at the 2Rb and/or 3Ra paracentromeric chromosome inversions. There was a non-random distribution of cattle-fed mosquitoes between the PCA clusters, suggesting that alleles linked to the 2Rb and/or 3Ra inversions may influence host choice. Using a novel inversion genotyping assay, we detected a significant enrichment of the standard arrangement (non-inverted) of 3Ra among cattle-fed mosquitoes (N = 129) versus all non-cattle-fed individuals (N = 234; χ^2^, *p* = 0.007). Thus, tracking the frequency of the 3Ra in *An*. *arabiensis* populations may be of use to infer selection on host choice behavior within these vector populations; possibly in response to vector control. Controlled host-choice assays are needed to discern whether the observed genetic component has a direct relationship with innate host preference. A better understanding of the genetic basis for host feeding behavior in *An*. *arabiensis* may also open avenues for novel vector control strategies based on driving genes for zoophily into wild mosquito populations.

## Author Summary

Malaria transmission is driven by the propensity for mosquito vectors to bite people, while its control depends on the tendency of mosquitoes to bite and rest in places where they will come into contact with insecticides. In many parts of Africa, where coverage with Long Lasting Insecticide Treated Nets is high, *Anopheles arabiensis* is the only remaing malaria vector. We sought to assess the potential for *An*. *arabiensis* to adapt its behavior to avoid control measures by investigating the genetic basis for its host choice and resting behavior. Blood fed *An*. *arabiensis* were collected resting indoors and outdoors in the Kilombero Valley, Tanzania. We sequenced a total of 48 genomes representing 4 phenotypes (human or cow fed, resting in or outdoors) and tested for genetic associations with each phenotype. Genomic analysis followed up by application of a novel molecular karyotyping assay revealed a relationship between *An*. *arabiensis* that fed on cattle and the standard arrangement of the 3Ra inversion. This is strong support that *An*. *arabiensis* blood-feeding behavior has a substantial genetic component. Controlled host choice assays are needed to confirm a direct link between allelic variation within the 3Ra inversion and host preference.

## Introduction

Blood-feeding insects impose a substantial burden on human and animal health through their role as disease vectors. In particular, mosquito species that feed on human blood pose an enormous public health threat by transmitting numerous pathogens such as dengue virus, Zika virus and malaria, which together kill more than one million people per year [1,2]. Human exposure to pathogens transmitted by mosquito vectors is determined by vector behaviors such as: (1) propensity to feed on humans relative to other animals (anthropophily) and (2) preference for living in close proximity to humans, as reflected by biting and resting inside houses (endophily) [3]. These traits are known to vary within and between the *Anopheles* mosquito species that transmit malaria [3]. It has been demonstrated since the earliest days of malaria transmission modeling that the degree of anthropophily in vector populations is strongly associated with transmission intensity [4]. Furthermore, the extent to which vectors feed and rest inside houses is a critical determinant of the effectiveness of current frontline control strategies including Long-Lasting Insecticide Treated Nets (LLINs) and Indoor Residual Spraying (IRS), which selectively kill mosquitoes that bite and rest indoors [1].

Vector species with a relatively broad host range, like *Anopheles arabiensis*, are thought to be better able to persist in areas of high indoor insecticide use. This is because they are more likely to avoid feeding and resting in areas protected by insecticides. For example, several studies in East Africa have shown dramatic declines in the abundance of the highly anthropophilic species *An*. *gambiae* s.s. relative to *An*. *arabiensis* as LLIN usage has increased [5–13]. Similar changes in vector species composition in response to LLINs have been reported outside of Africa, including in the Solomon Islands where the highly endophagic and anthropophilic *An*. *punctulatus* has been nearly eliminated by LLINs whereas the more exophilic *An*. *farauti* remains [14]. Given the importance of mosquito feeding and resting behavior to the effectiveness of malaria control and transmission, there is an urgent need to understand the underlying biological determinants of these behaviors and their short and long term impact on the effectiveness of the existing frontline interventions.

Environmental heterogeneity is known to have a substantial influence on several important vector behaviors [15], including host choice and resting behavior [3]. For example, a recent study in southern Tanzania reported that the proportion of blood meals taken from humans by *An*. *arabiensis* fell by over 50% when at least one cow was kept at a household [16]. The resting behavior of mosquito vectors in this study was also highly associated with proximity to livestock; the proportion of *An*. *arabiensis* resting indoors fell by ~50% when cattle were present at the household [16]. While these studies demonstrate that the environment can influence *An*. *arabiensis* behavior, far less is known about the influence of mosquito genetics on these behavioral phenotypes. An early study by Gillies [17] experimentally investigated the potential heritability of host choice behavior in *An*. *gambiae* s.l., and showed these vectors significantly increased their preference for cattle hosts (relative to humans) within a few generations of selection. Other early work demonstrated an association between the 3Ra chromosomal inversion and *An*. *arabiensis* found in cattle-sheds [18], and between the 2Rb chromosomal inversion and human-feeding [19]. Understanding the genetic basis for host choice behavior is essential for elucidation of the co-evolutionary forces that stabilize the transmission of vector-borne diseases, and may enable the development of genetic markers that could be used for rapid quantification of the degree of anthropophily in vector populations as is required to estimate transmission risk and plan vector control [20].

In the dengue and zika vector, *Aedes aegypti*, allelic variation in the odorant receptor gene *Or4* has been linked to human-biting preference [21]. However, to date, no ortholog for *AaegOr4* has been identified in Anopheline mosquitoes [22], and no direct functional links between genetic mutations in African malaria vectors and behaviors that influence transmission potential have been identified [3,23–25]. As the genera *Aedes* and *Anopheles* diverged before the emergence of humans (~150MYA) [26], anthropophily likely evolved independently in these species and may involve distinct mechanisms. Developing the ability to track mosquito behaviors such as biting time [27], degree of anthropophily [3], and resting behavior [28] will help improve vector surveillance and anticipation of whether the effectiveness of control measures are being eroded by mosquito behavioral adaptations [29]. Shifts in mosquito behavior that reduce their contact with interventions, termed behavioral avoidance, may be a significant threat to the long-term goal of malaria elimination [30]. Thus, understanding the genetic contribution to these phenotypes is a critical first step toward effective mosquito control in the future.

Due to the role of *An*. *arabiensis* in maintaining residual malaria transmission across much of sub-Saharan Africa [8,13,31], we conducted a field survey to elucidate the genetic basis of host preference and resting habitat choice in this phenotypically variable species. This is the first application of both whole genome sequencing and a population-scale assessment of chromosome inversion frequencies to test for associations between Anopheles mosquito behavioral phenotypes and genotype. As a proxy for host preference and resting behavior, we identified which host each field-collected mosquito fed upon and noted whether it was collected indoors or outdoors. We sampled hundreds of individuals to overcome phenotypic plasticity due to environmental factors (i.e. incomplete penetrance). Assaying these phenotypes in nature is important because lab experiments do not always translate to the field. However, we cannot be certain which choices a given mosquito had before collection, beyond the knowledge that humans and livestock were present in the village of collection. We also cannot conclude causation based on association alone, as other factors unrelated to host preference may be influencing allele frequencies among subpopulations (e.g. insecticide resistance). These limitations should be kept in mind when phenotypes are described in this study.

## Results

### Analysis of host choice

**Fig 1:**
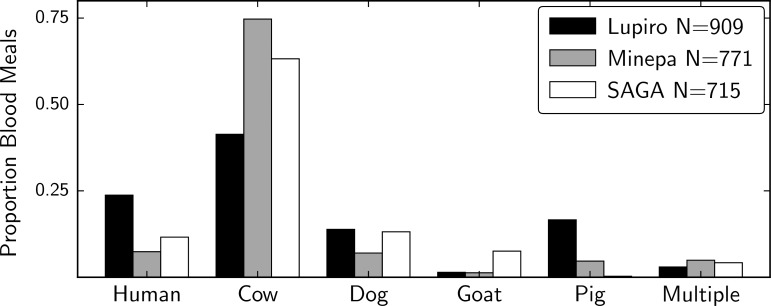
Relative host choice between villages. This figure describes the results of bloodmeal analysis of *An*. *arabiensis* collected from: Lupiro, Minepa, and Sagamaganga (SAGA). We detected multiple hosts in <5% of individuals. Different combinations of mixed host bloodmeals were pooled and shown as “Multiple”. A few chicken bloodmeals were also detected at each site.

We analyzed the blood meals from 1,731 *An*. *arabiensis* females that were captured resting indoors or outdoors in 3 villages in Tanzania. Specific hosts were identified using a multiplex genotyping assay performed on DNA extracted from female abdomens (see Materials and Methods). The relative frequencies of different host species in blood meals varied by site, but cattle was the most abundant blood source detected in all three. Lupiro had a significantly higher proportion of human-fed mosquitoes (24%; *P* <0.0001, Fisher exact) compared to Minepa (7%) and Sagamaganga (11%; Fig 1 and S1 and S2 Tables). Mosquitoes that tested positive for more than one host were rare (<5%; Fig 1). To investigate temporal and spatial variation of host choice, mosquitoes were collected from several households throughout a period of 2 years (S9 Table). A backward elimination model selection approach using a Generalized Linear Mixed Model (GLMM) was used to investigate whether host choice was impacted by different environmental factors. Livestock presence at the household level, season (dry or wet), village and trapping location (indoors or out) were included into a maximum model as fixed effects, while collection date and household were added as random effects (S10 Table). The final model showed that livestock presence at the household level and trapping location (indoor or outdoor) were associated with the frequency of human fed mosquitoes found. The proportion of human fed *An*. *arabiensis* was inversely correlated with the presence of livestock (*P*<0.0001, Coeff = -1.92; GLMM, S11 Table). The frequency of human fed mosquitoes was also higher in indoor vs outdoor collections (*P* = 0.0083, Coeff = -0.7349; GLMM, S11 Table).

### Testing for a genetic component underlying host choice and indoor resting behavior

We sequenced a total of 48 individual *An*. *arabiensis* genomes (median coverage = 18x; S3 Table). In terms of host choice, this collection included 25 cattle-fed and 23 human-fed individuals from both indoor (N = 24) and outdoor (N = 24) resting sites. From these genomes, we identified a set of 4,820,851 segregating SNPs after a minor allele frequency threshold of 10% was imposed. Using these data, we estimated the genetic component (or “SNP heritability” [32]) for each phenotype (see Materials and Methods). The heritability estimate for human-fed vs. cattle-fed mosquitoes was *H*^2^ = 0.94, SE = 3.47 and indoor vs. outdoor was *H*^2^ = 0.05, SE = 2.34. Due to high error estimates, we permuted the phenotypes to simulate the null hypothesis of no connection between the SNP data and each behavior. We then compared the estimate of the SNP heritability from the real data with the estimates from each of 10,000 permutations. This test supports the initial heritability estimates indicating a genetic component for host choice (human vs. cow fed; permuted *P* = 0.001) and no genetic component for resting behavior (indoor vs. outdoor, permuted *P* = 0.470). Due to the lack of evidence for a genetic component for resting behavior, we restricted further analysis to elucidating the observed association between host choice and genotype.

### Genetic structure

**Fig 2:**
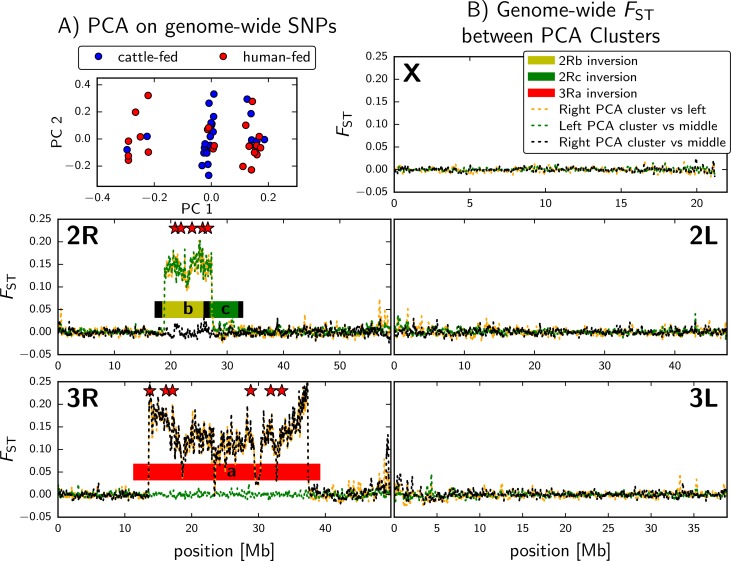
Genetic variation explained by the 2Rb and 3Ra inversions. a) Genetic structure was assessed using genome-wide SNP data for individual *An*. *arabiensis* females using a PCA analysis. Three discrete PCA clusters were observed. Red = human-fed and blue = cattle-fed. There is an enrichment of cattle-fed individuals in the middle PCA cluster (P < 0.001; Fisher Exact). (b) To reveal differentiated genomic regions underlying the distinct PCA clusters (left, middle, and right) we plotted F_ST_ for each chromosome in 100kb windows with 20kb steps between the PCA clusters. The outside PCA clusters differed at the 2Rb and 3Ra inversions (orange), left versus middle PCA clusters differed at 2Rb only (green), and right versus middle differed at 3Ra only (black). Stars indicate the position of SNPs chosen for the inversion genotyping assay.

To test for the existence of genetic structure within our set of 48 sequenced genomes, individuals were partitioned by genetic relatedness using a Principle Component Analysis on all SNPs (PCA; see methods). Using this approach, we observed 3 discrete genetic clusters (Fig 2A). Genome-wide *F*_*ST*_ in sliding windows between individuals in each PCA cluster revealed that the clusters can be explained by distinct combinations of 3Ra and 2Rb chromosome inversion states (Fig 2B). Using a novel inversion genotyping assay (see Materials and Methods), we determined the 2Rb and 3Ra inversion states for individuals represented in each PCA cluster (2Rb_3Ra): left = bb_a+, middle = bb_++, and right = b+_++. There was an enrichment of cattle-fed mosquitoes among bb_++ individuals (*P* < 0.001; Fisher Exact with Freeman-Halton extension).

### Testing for associations between inversion state and host choice

To explore the relationship between the 3Ra and 2Rb inversion state and host choice, we developed and employed an inversion genotyping assay. In brief, we selected SNPs near the inversion breakpoints (Fig 2B) with extreme *F*_*ST*_ values between genomes grouped by distinct 3Ra or 2Rb inversion states. We then genotyped samples for our 11 inversion diagnostic SNPs (6 for 3Ra and 5 for 2Rb) in parallel using the Sequenome iPLEX platform (see Materials and Methods). We associated genotype information with the standard or inverted arrangement of the 2Rb and 3Ra inversions by genotyping karyotyped samples (S5 Table). In total, we genotyped 363 bloodfed females from the village of Lupiro for inversion state (S6 Table). The samples were composed primarily of human-fed (37%) or cattle-fed mosquitoes (36%; S7 Table). The 2Rb and 3Ra inversion frequencies were within Hardy-Weinberg (HW) expectations for all samples (*P* = 0.55 and 0.90, respectively). However, the 3Ra inversion was outside of HW among dog-fed individuals (*P* = 0.02; N = 40, S7 Table). Only four 3Ra homozygotes were observed (N = 363); three fed on dog and one fed on human. The frequency of the 3Ra inversion in Lupiro ranged from 7.94% in cattle to 16.67% in pig-fed mosquitoes. The 2Rb inversion ranged from 81.06% in human to 95% in dog-fed specimens (Table 1). We focused on three major comparisons to test for a relationship between inversion state and host choice: 1) cattle-fed versus human-fed, 2) human-fed versus non-human-fed, and 3) cattle-fed versus non-cattle-fed. After correcting for multiple tests (significant *p-value* threshold = 0.017), there was no evidence for an enrichment of the standard arrangement of 3Ra (3R+) in cattle-fed mosquitoes compared to human-fed (*P* = 0.047, χ^2^; N = 263; Table 1) and no relationship was detected between 3Ra and human-fed versus non-human-fed mosquitoes (*P* = 0.553, χ^2^; N = 263; Table 1). However, a significant enrichment of the standard arrangement of 3Ra (3R+) was observed in cattle-fed versus non-cattle-fed (*P* = 0.007, χ^2^, N = 363; Table 1).

**Table 1:** 3Ra and 2Rb Inversion frequencies by host.

**Host**	**++**	**a+**	**aa**	**N**	**a**	**+**	**freq a**
**human**	99	32	1	132	34	230	12.88%
**cattle**	106	20	0	126	20	232	7.9%
**pig**	38	19	0	57	19	95	16.67%
**dog**	30	7	3	40	13	67	16.25%
**goat**	2	1	0	3	1	5	
**cattle+goat**	2	0	0	2	0	4	
**human+cattle**	1	0	0	1	0	2	
**dog+human**	0	1	0	1	1	1	
**dog+pig**	1	0	0	1	0	2	
				**human**	35	233	13.06%
				**non-human**	53	405	11.57%
				**cattle**	20	238	7.75% *
				**non-cattle**	68	400	14.53%
							
**Host**	**++**	**b+**	**bb**	**N**	**b**	**+**	**freq b**
**human**	4	42	86	132	214	50	81.06%
**cattle**	4	33	89	126	211	41	83.73%
**pig**	1	18	38	57	94	20	82.46%
**dog**	0	4	36	40	76	4	95.00%
**goat**	0	1	2	3	5	1	
**cattle+goat**	0	1	1	2	3	1	
**human+cattle**	0	0	1	1	2	0	
**human+dog**	0	1	0	1	1	1	
**dog+Pig**	0	0	1	1	2	0	
				**human**	217	51	80.97%
				**non-human**	391	67	85.37%
				**cattle**	216	42	83.72%
				**non-cattle**	392	76	83.76%

Table 1: Mosquitoes were collected from the village of Lupiro. The inversion frequencies (freq a or b) were not calculated for host categories with low sample sizes. Note the significantly lower frequency of 3Ra among cattle-fed mosquitoes (*). The sum of human- and cattle-fed mosquitoes (bottom four categories) included pure (e.g. human) and mixed host (e.g. dog+human) samples.

### Candidate genes within 3Ra

Due to the association between host choice and 3Ra, we explored allelic variation in genes in the “odorant binding” gene ontology category (GO:0005549) that occur within the 3Ra breakpoints. To accomplish this, we sorted “odorant binding” genes by mean *F*_*ST*_ estimates for each gene (plus 1kb upstream) between 3Ra standard (N = 39) and 3Ra inverted (N = 9) genomes (S8 Table). Among the genes with the highest *F*_*ST*_ was odorant binding protein antennal (*Obp5* in *An*. *gambiae*; 5^th^ highest mean *F*_*ST*_ = 0.2) and the odorant receptor *Or65* (10^th^ highest mean *F*_*ST*_ = 0.18; S8 Table).

## Discussion

In this study, we investigate the genetic basis of host choice and resting behavior in *An*. *arabiensis* using whole genome sequencing and a novel chromosomal inversion genotyping assay. We did not detect a genetic component (“SNP heritability”) for resting behavior (endo- versus exo-phily). This could be due to substantial “behavioral plasticity” in this phenotype [33,34]. A genetic component for host choice was detected through analysis of genome-wide SNP data. Population-scale inversion genotyping revealed an association between the standard arrangement of 3Ra (3R+) and cattle-fed *An*. *arabiensis*. Identifying functional alleles underlying host choice in *An*. *arabiensis* has relevance because this species has become the dominant malaria vector in many parts of East Africa, where insecticide use is common [13,35–37]. We highlight two intriguing candidate genes within the 3Ra, including the odorant binding protein *Obp5*, and the odorant receptor *Or65*. *Obp5* is prominently expressed in female antennae and is significantly overexpressed in female versus male heads [38]. Thus, *Obp5* may be involved in host seeking behavior. *Obp5* is also significantly overexpressed in non-bloodfed females compared to those who have taken a blood meal in the previous 24 hours [39], further implicating its importance in host seeking behavior. We also detected allelic variation in *Or65* between 3Ra inversion arrangements. This gene is a “highly tuned” odorant receptor, that has been shown to be responsive to 2-ethylphenol, a compound found in animal urine [40]. This analysis resulted in some compelling candidate genes, which may be involved in host detection and host discrimination. Controlled host preference assays with distinct genotypes are needed to test for functional links between these candidate genes, among others, and host choice.

“SNP heritability” provides an estimate of the correlation between phenotype and genome-wide SNP profile [32]. A strength of this metric is its robustness to detect genetic components for complex phenotypes that are influenced by many small-effect mutations, which may be the case for host choice in *An*. *arabiensis*. In this study, we collected mosquitoes that were blood-fed and resting either indoors or outdoors to assess the genetic basis of host choice and resting behavior. Each phenotype is complex and may be affected, at least in part, by innate preference and the local environment, including the availability of hosts and indoor resting sites. Despite our inability to control for environmental heterogeneities in the field, the SNP heritability analysis detected a genetic component for host choice. Due to the low LD (~200bp) across the genome of this species [41], larger samples sizes (e.g. 100–1000) are needed to rigorously quantify the SNP heritability of host choice, and potentially identify additional candidate genes. Larger sample sizes may also uncover a genetic component to resting behavior, which we did not detect here but cannot rule out. Previously, high inversion polymorphism has been detected in *An*. *arabiensis* in Nigeria with some inversions showing changes in frequencies linked to different geographical areas [42]. Hypothesizing there is a functional link between 3Ra and host choice, changes in inversion frequencies could be driven by a higher relative fitness for cattle-biting genotypes in areas with high LLIN usage and/or lower relative fitness of cattle-biting mosquitoes in areas with low cattle density.

This analysis of the genetic basis of host choice in *An*. *arabiensis* revealed an association between 3R+ and cattle-feeding. Previously, indirect associations have also been made between host choice and inversions, like the 3Ra in Ethiopia [18] and Kenya [43]. A non-random distribution of the 2Rb inversion has also been reported between human- and cattle-fed mosquitoes [19], but our study is the first to analyze paired karyotype and host choice information from each individual mosquito. Thus, our multiplex genotyping assays allowed us to directly estimate relationships between host choice and genotype in wild mosquitoes in a high-throughput and economical fashion. To ensure that our genotyping method was robust, we selected multiple SNPs near the inversion breakpoints for each inversion. It should be noted that each inversion state represents a suite of linked alleles located primarily within the inversion breakpoints. Further testing is needed to assess how well this assay would perform on *An*. *arabiensis* samples from outside our study sites in Tanzania.

The enrichment of 3R+ among cattle-fed mosquitoes provides support for a genetic component to host choice, which is consistent with the report that zoophily can be selected for [17]. The elevated frequency of the 3Ra inversion among various hosts, including pig-fed, dog-fed, goat-fed, and human-fed mosquitoes is suggestive that 3Ra individuals are less choosey. There is also an enrichment of 3Ra/a homozygotes among dog-fed mosquitoes, which is interesting because these genotypes are sufficiently rare in Tanzania that some have even postulated the presence of a recessive lethal in 3Ra [44]. The fact that all other species in the *Anopheles gambia*e species complex are fixed for the standard arrangement of 3Ra, strongly suggests that 3Ra is derived [45]. Thus, one possible explanation for the observed results is that 3R+ is the ancestral state and alleles therein facilitate specialization on cattle. A loss-of-function mutation in one or more of these genes could then have been acquired early on in the haplotype representing the inverted arrangement of 3Ra, resulting in an expanded host range. This hypothesis is consistent with behavioral heterogeneities and 3Ra frequencies across Africa. For example, *An*. *arabiensis* is reportedly more anthropophilic in West African countries like Burkina Faso and Mali [46,47], where the frequency of 3Ra is very high (~40–60%; [48–51]) compared to East African populations, like our tudy area in Tanzania (~12% or less), and others [18,43,52–54]. The diversity of host feeding behaviors among species in the *An*. *gambiae* complex, including extreme host specialists (e.g. *An*. *gambaie s*.*s*.) and those with wider host ranges (e.g. *An*. *arabiensis* with 3Ra), make this a fascinating system to study the evolution of host choice.

While we provide strong evidence for a role of allelic variation within 3Ra underlying *An*. *arabiensis* host choice, the effect size (i.e. relative contribution to the phenotype) is unclear. Controlling for environmental variation is likely to be very important when choosing fully representative samples for each phenotype. For example, a human-fed mosquito may provide a more meaningful representation of host preference if there is an abundance of alternative hosts nearby (e.g. cattle). Some populations of *An*. *arabiensis* persistently bite people despite being surrounded by cattle [55]. This highlights the importance of integrating genetic analyses into the wider ecological context in which behavioral phenotypes can be expressed. In a field study like ours, the host choice phenotype measured is a product of both the availability of different host species, and a mosquito’s innate preference for them. We attempted to account for ecological factors like cattle and human host availability, but each host may not have been equally accessible to mosquitoes due to factors such as the use of bednets by humans, insecticides on cattle, or other barriers to choice that are not perceptible to observers. While field studies are invaluable first steps to detect genetic components to important phenotypes, more detailed experimental manipulations will be required to confirm the role of alleles within the 3Ra inversion on blood feeding behavior. For this, we advocate laboratory or semi-field assays in which groups of mosquitoes from each 3Ra inversion state are given a direct choice between different host types in a controlled environment.

This is the first study to report a genetic component to host choice behavior in the major malaria vector *An*. *arabiensis* and we link this behavior to allelic variation between the 3Ra inversion states. Mosquitoes that had fed on cattle were significantly more likely to have the presumably ancestral 3R+ inversion. Given that human feeding is essential for malaria transmission, these results may help identify specific markers for assessing the transmission potential of vector populations, and how their behavior evolves in response to control measures, such as insecticide treated nets, which selectively kill mosquitoes attempting to feed on people. This association and the introduction of a novel inversion genotyping assay may be a valuable tool for future malaria vector surveillance. For example, tracking the frequency of the 3Ra inversion in *An*. *arabiensis* may elucidate the emergence of behavioral avoidance (e.g. shifting toward zoophily) so countermeasures can be implemented. A better understanding of the genetic basis for host choice in *An*. *arabiensis* may also improve vector control if cattle-biting mosquitoes can be genetically engineered and released in the population, having an effect similar in concept to zooprophylaxis [56].

## Materials and Methods

### Mosquito collection area

The mosquitoes were collected within 3 villages in the Kilombero River Valley in south-eastern Tanzania: Lupiro (S08°23.2956'; E036°40.6122'), Minepa (S08°16.4974'; E036°40.7640') and Sagamaganga (S08°03.8392'; E036°47.7709'). The Kilombero Valley is dominated by irrigated and rain-fed rice paddies and maize fields bordered by woodland. The annual rainfall is 1200–1800 mm with two rainy seasons. The average daily temperatures range between 20°C and 33°C. Most people in this area are subsistence farmers and/or livestock keepers. Mud or brick houses stand in clusters among a few trees. If a household owns livestock, the animals are kept outside a few meters away from the house in sheds (pigs and goats) or within cattle fences. Animal sheds with walls and a roof were considered indoor resting areas. Inside houses you will regularly find chickens, cats and sometimes dogs. The mosquitoes will encounter bed nets inside almost all houses in the valley, but repellents are rarely used by people outdoors [57] and livestock are not treated with insecticide [58]. Malaria is endemic in these communities and although prevalence is declining, almost all inhabitants have antibodies for the disease [59]. The dominant malaria vector species are *An*. *arabiensis* and the *An*. *funestus* group [60].

### Collection methods

In each village, households chosen for collection were within 100-200m of one another. Indoor mosquito collection method was aspiration using a standard battery-powered CDC Back Pack aspirator (BP, Model 1412, John Hock, Florida USA) [61]. In these collections, the aspirator was used to collect mosquitoes from the main bedroom by sweeping the nozzle over the interior walls, roof and furniture for a fixed period of ten minutes. BP collections were timed to standardize sampling effort across houses. A resting bucket trap (RBu) was used to trap mosquitoes outdoors. The RBu is made from a standard 20 liter plastic bucket lined with black cotton cloth, and set by placing it on its side with the open end facing a house at a distance of approximately 5m. A small wet cloth is placed inside the bucket to increase humidity. Mosquitoes resting inside RBus were collected at dawn by placing the nozzle of a battery-powered modified CDC backpack aspirator at the open end of the bucket and aspirating for 10–20 seconds.

### Ethics

Before collection, meetings were held with community leaders in all villages during which they were informed about the purpose of the study and their participation requested. After their permission had been granted, the study team visited each village and informed consent was obtained from each head of household where trapping was conducted. Research clearance was obtained from the institutional review board of Ifakara Health Institute in Tanzania (IHI/IRB/No: 16–2013) and by the National Institute for Medical Research in Tanzania (NIMR/HQ/R.8c/Vol. II/304).

### DNA extraction

For each specimen, the abdomen was separated from the head and thorax and DNA was extracted separately from each using the QIAGEN Biosprint 96 system and QIAGEN blood and tissue kits (QIAGEN, Valencia, CA). *Anopheles arabiensis* samples were distinguished from other *An*. *gambiae* s.l. species complex members with the Scott polymerase chain reaction assay [62] and their DNA content was quantified using the Qubit 2.0 Fluorometer (Life technologies, Grand Island, NY).

### Bloodmeal analysis

The specific host species that each mosquito had fed upon was determined by a multiplex genotyping assay on DNA extracted from abdomens [63]. This multiplex genotyping assay can distinguish between blood from cattle, goat, pig, dog, chicken and human.

### Analysis of host choice

Statistical analysis was conducted to compare the proportion of human-fed mosquitoes between villages and between resting habitats (indoors vs outdoors) using the statistical software R (Core-Team RD, 2013). Variation in the proportion of human-fed *An*. *arabiensis* within the total catch was investigated. Samples found to contain any human blood represented one category and those containing animal blood another. Generalized linear mixed effects models (GLMM, package lme4 in R [64]) were used, with human-fed mosquitoes versus animal-fed mosquitoes as a response variable with a binomial distribution and fitting village and livestock presence as fixed effects, and date and house of collection as random effects. To explore the resting behavior of *An*. *arabiensis* as a response variable, only mosquitoes resting in houses or outdoors but not those caught resting in animal sheds were used for analysis. Here the GLMM were fitted for each village separately with human-fed mosquitoes caught indoors versus outdoors as a response variable with a binomial distribution and livestock as fixed effect and date and house of collection as random effects.

### Cytogenetic analysis

To identify 3Ra, 2Rb, and 2Rc chromosomal inversions, polytene chromosomes were extracted from ovarian nurse cells from half gravid indoor resting mosquitoes using the protocol described by Hunt [65]. Chromosome banding patterns were examined using a Nikon Eclipse e600 phase contrast microscope. The genotypes of the chromosome inversions were scored for each individual mosquito. Photographic images of chromosomes for the majority of karyotyped individual mosquitoes used in this study are available on PopI OpenProject page—AaGenome (https://popi.ucdavis.edu/PopulationData/OpenProjects/AaGenome).

### Genomic library preparation and sequencing

To avoid identifying SNPs associated with demography or other environmental factors, we chose to sequence mosquitoes collected from only one village, Lupiro. We focused on this village because it had sufficient human-fed mosquitoes for testing (Fig 1). Genomic DNA was quantified using a Qubit 2.0 fluorometer (Life Technologies). We used 25-50ng of input DNA for library construction. DNA was then cleaned and concentrated with the DNA Clean and Concentrator kit (Zymo Research Corporation). Library preparations were made with the Nextera DNA Sample Preparation Kit (Illumina) and TruSeq dual indexing barcodes (Illumina). Libraries were size-selected with Agencourt AMPure XP beads (Beckman Coulter). We assessed the insert size distribution of the final libraries using a QIAxcel instrument (Qiagen, Valencia, CA) or Bioanalyzer 2100 (Agilent), and the final library concentration was measured with a Qubit 2.0 fluorometer (Life Technologies). Individually barcoded libraries were sequenced with the Illumina HiSeq2500 platform with paired-end 100 base pair reads, at the QB3 Vincent J Coates Genomics Sequencing Laboratory at UC Berkeley. See S3 Table for sequence depth information for each sample.

### Genome sequence mapping and SNP identification

We assessed the quality of our genome sequencing reads using the FastQC software (http://www.bioinformatics.babraham.ac.uk/projects/fastqc/). Adaptor sequences and poor quality sequence were trimmed from the raw Illumina Fastq reads using the Trimmomatic software, version 0.30 [66], with default options. Reads were aligned with BWA-mem [67] to the assembled *An*. *arabiensis* reference genome version AaraCHR (generously provided by Xiaofang Jiang, Brantley Hall, and Igor Sharakhov. Also see [68]). We used the MarkDuplicates module from Picard tools to remove PCR duplicates and the Genome Analysis Tool Kit (GATK) v1.7 to realign reads around indels [69]. The resulting sorted BAM (Binary sequence Alignment/Map) files containing sequences for each read and its mapping position were then used to make a VCF (Variant Call Format) file using samtools (v1.1–12) ‘mpileup’ and bcftools (v1.1–36) multiallelic-caller. We removed indels using VCFtools (v0.1.13; “—remove-indels”) and filtered for variable sites using a minor allele frequency threshold of 0.10 (“—maf 0.1”) and a major allele threshold of 0.9 (“—max-maf 0.9”).

### Estimating SNP heritability of each phenotype

Host choice and resting behavior phenotypes may be influenced by many small-effect mutations across the genome. SNP heritability is the correlation between the genome-wide genotypic variation and phenotypic variance (V(G) / V(p)). To estimate SNP heritability, the VCF file containing genome-wide SNP data for all samples was converted to PLINK with VCFtools (command “vcftools—plink”) and then binary ped files (GCTA option: “—make-bed”) for analysis with the Genome-Wide Complex Trait Analysis software (GCTA; [70]). To calculate “SNP heritability” with GCTA, we first generated a genetic relationship matrix. Then we calculated SNP heritability for host choice (estimated human-fed prevalence = 20%) and resting behavior (estimated indoor prevalence = 43%). To estimate the permuted p-value, we used a custom python script to randomly permute the phenotype key for 10000 iterations. The permuted p-value was estimated from the proportion of heritability estimates from the randomly permuted phenotype key that were greater than the heritability estimate from the real data.

### Chromosomal inversion genotyping assay

We used GCTA [70] to perform a principal component analysis (PCA) on all whole genome sequenced individuals from Lupiro. This partitioned the individuals into at least three clusters. Genomic differentiation among the three clusters was concentrated in regions corresponding to 2Rb and 3Ra inversions (Fig 2). We identified candidate diagnostic SNPs between the three clusters using F_ST_ values. We selected 6 diagnostic SNPs for 3Ra that span 19.76Mbp, and 5 diagnostic SNPs for 2Rb spanning 6Mbp (Fig 2B). A multiplex SNP genotyping assay was designed for an iPLEX assay platform using Sequenom Typer AssayDesigner program (S4 Table). The Veterinary Genetics Laboratory at UC Davis performed genotyping using the Sequenom iPLEX platform.

### Data accessibility

The sequencing data have been uploaded to NCBI's Sequence Read Archive (SRA) under project accession number SRP077062 (http://trace.ncbi.nlm.nih.gov/Traces/study/?acc=SRP077062&go=go). Additional meta data associated with this study are available on the open source online vector database PopI: AaGenome (https://popi.ucdavis.edu/PopulationData/OpenProjects/AaGenome/).

## Supporting Information

S1 Table Blood meal analysis by site. This is a table of all wild-caught *An*. *arabiensis* with associated metadata.
(XLSX)

S2 Table Blood meal summary. This table summarizes the frequencies of each host that were detected in wild-caught *An*. *arabiensis* bloodmeals at the field sites of Lupiro, Minepa, Sagamaganga, and overall. Lupiro had a higher proportion of human-fed mosquitoes compared to Minepa and Sagamaganga (*P* < 0.01).
(XLSX)

S3 Table Individual *An*. *arabiensis* genomes from the village of Lupiro. This table lists each *An*. *arabiensis* sample that was sequenced (whole-genome) and includes associated metadata for each.
(XLSX)

S4 Table Inversion genotyping iPLEX primers. This table lists the primer sequences used for the multiplex inversion genotyping assay. These primers target 6 3Ra diagnostic SNPs that span 19.76Mbp, and 5 diagnostic SNPs for 2Rb spanning 6Mbp (Fig 2B).
(XLSX)

S5 Table Inversion genotype polarization (standard vs. inverted). This table shows the results of the inversion genotyping assay on karyotyped samples collected from our field sites. High LD between markers spanning the 2Rb or 3Ra inversions enabled confident differentiation of the distinct inversion states. These results were used to polarize of the genotype calls to either the standard or inverted arrangement.
(XLSX)

S6 Table Inversion genotyping results. This table contains all the inversion calls and associated genotype data matched with bloodmeal data.
(XLSX)

S7 Table Genotyping data summary. These tables summarize the inversion and host data. The frequency of the 3Ra was much lower among cattle-fed (7.75%) versus non-cattlefed (14.53%) mosquitoes. Both the 2Rb and 3Ra were within Hardy-Weinberg expectations, except for the dog-fed subgroup, which had an enrichment of 3Ra homozygotes (*P* = 0.02).
(XLSX)

S8 Table Candidate "odorant binding" genes in 3Ra. To highlight candidate genes that may influence host preference we collected all genes within the “odorant binding” gene ontology category (GO:0005549) that occur within the 3Ra inversion breakpoints. Then, we sorted the genes based on mean *F*_*ST*_ estimates for each gene (plus 1kb upstream to include regulatory variation) between 3Ra standard (N = 39) and 3Ra inverted (N = 9) genomes. We chose to focus on odorant binging genes because the odorant receptor *Or4* was found to be involved in host seeking behavior in *Aedes aegypti* [21]. This approach assumes that the genes that are the most diverged between inversion states are more likely to have functional differences. Additionally, this method excludes non-odorant binding genes, which might also influence host preference.
(XLSX)

S9 Table Sample collection dates and households visited. This table summarizes the collection dates and how many houses were visited.
(XLSX)

S10 Table Modeling environmental effects. This table shows the results of the maximum model used to test for environmental effects on each phenotype. The model shows that the presence of livestock at the household level and trapping location (indoor or outdoor) were associated with the frequency of human-fed mosquitoes.
(XLSX)

S11 Table Environmental modeling results. This table summarizes the results from the generalized linear mixed model (GLMM), which tested for environmental effects on host preference and resting behavior. The proportion of human-fed *An*. *arabiensis* varied by household and was inversely correlated with the presence of livestock (*P*<0.0001). The frequency of human fed mosquitoes was also correlated with trapping location–less human-fed mosquitoes were collected in outdoor traps (*P* = 0.0083).
(XLSX)
